# Effect of Vacuum Level and Pulsation Parameters on Milking Efficiency and Animal Welfare of Murciano-Granadina Goats Milked in Mid-Line and Low-Line Milking Machines

**DOI:** 10.3390/ani12010040

**Published:** 2021-12-25

**Authors:** Gema Romero, Joel Bueso-Ródenas, Manuel Alejandro, Francisco Moya, José Ramón Díaz

**Affiliations:** 1 Departamento de Tecnología Agroalimentaria, Universidad Miguel Hernández (UMH), Ctra. de Beniel km 3.2, 03312 Orihuela, Spain; gemaromero@umh.es; 2 Departamento de Producción Animal y Salud Pública, Universidad Católica de Valencia (UCV), C/Guillem de Castro 94, 46001 Valencia, Spain; joel.bueso@ucv.es (J.B.-R.); kpra@kpra.es (F.M.); 3 C/Anabel Segura, 7, 28108 Madrid, Spain; manuel_alejandromartinez@yahoo.com

**Keywords:** milk fractioning, milk cortisol, small ruminants, milking parameters

## Abstract

**Simple Summary:**

In recent years, several studies have been carried out to optimize milking efficiency in Murciano-Granadina goats, but to our knowledge there are no experiments combining different milking parameters (system vacuum, pulsation rate and pulsator ratio) in two different height level milking machines. In two short experiments, testing different combinations of the parameters mentioned, milk fractioning, milking efficiency, teat end status and animal welfare were analyzed. Results showed that the height of the milking machine pipes can have an influence on which parameters are most optimal for milking efficiency and animal welfare in the milking of Murciano-Granadina goats.

**Abstract:**

The Murciano-Granadina goat breed has been described as a slow milking breed. As milking machine parameters can affect milk extraction in terms of yield and time employed, two experiments of one-month duration were performed with 88 goats in Latin square design to find the best combination of these parameters. One of them was carried out in a mid-line milking machine and one in a low-line milking machine. For each of them, two vacuum levels (36 and 40 kPa), two pulsation rates (90 and 120 cycles/min) and two pulsator ratios (50 and 60%) were used and milking efficiency, sanitary status of the mammary gland, milk cortisol, and teat end status were evaluated. Results showed that in milking machines installed in mid- and low-line, the use of 40 kPa system vacuum, 60% pulsator ratio and 90 or 120 cycles/min pulsation rate achieved optimum milking fractioning and efficiency. In the case of low-level milking machines, a similar combination with 36 kPa not only showed worse milking fractioning values, but also provided better values of teat end status and cortisol level.

## 1. Introduction

Milking machine design and parameters can significantly affect milk extraction, in terms of quantity, quality and time employed in this task. Thus, in recent years, several studies have been focused on maximizing milking efficiency [1] or minimizing potential deleterious effects of machine milking on milk quality or sanitary status of the mammary gland, including teat end status [2,3], somatic cell count and incidence of mastitis [4,5]. Milking parameters, including vacuum level and pulsation rate and ratio, are known to affect animal welfare [6]. Minimum vacuum level values are required to open the teat sphincter, ensure stability of the teat cups on the animal and transport the milk along the milk line [7]. However, an excessive vacuum level can affect both the teat end condition and the mammary gland status [8]. On the other hand, pulsation limits teat end oedema and stimulates milk extraction [9]. In machine milking of goats, recommended settings are system vacuum level from 36 to 48 kPa, pulsation rate from 70 to 120 cycles/min and pulsator ratio of 60% [1,10,11]. 

**Figure 1:**
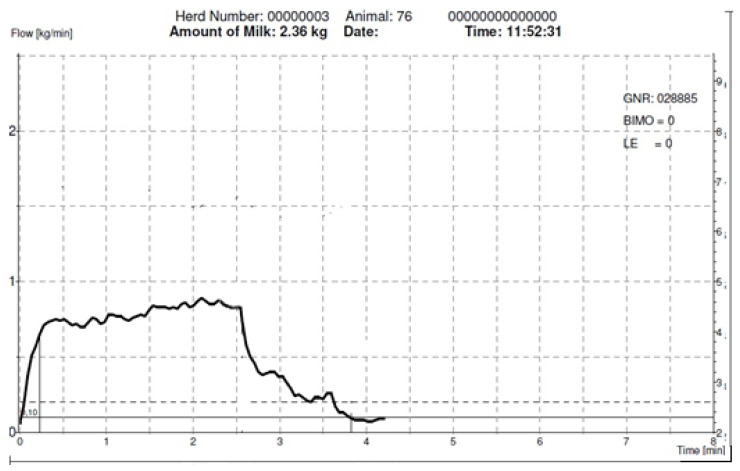
Milk emission curve recorded by the Lactocorder® devices (Lactocorder, Balgach, Switzerland) to show the emission curve that characterizes the Murciano-Granadina goat (long plateau phase).

**Figure 2:**
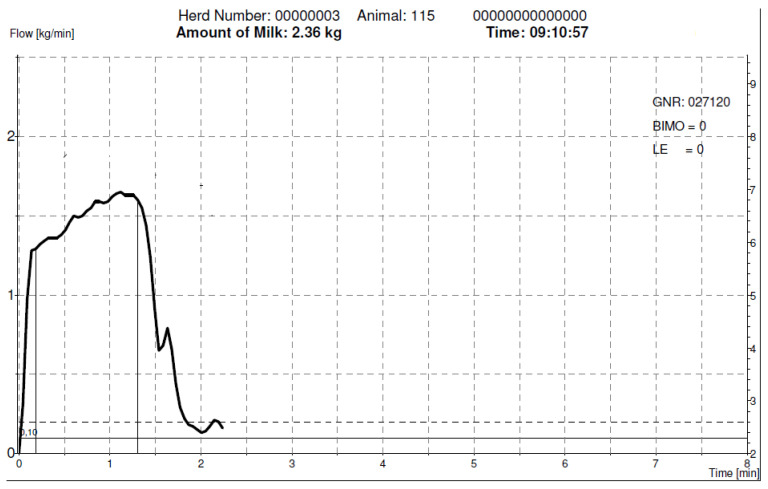
Milk emission curve recorded by the Lactocorder device to show the emission curve that characterizes other European breeds (high average milk flow).

The Murciano-Granadina goat breed has been described as a slow milking breed (Figure 1) when compared to other European breeds (Figure 2), such as Saanen. This difference has been related to udder characteristics, mainly teat sphincter strength [12]. Several recent studies have tested this breed in different conditions. In a short-term experiment carried out with a mid-line milking system. Fernandez et al. [13], found that the use of 42 kPa vacuum level combined with 120 cycles/min of pulsation rate improved milking efficiency when compared to 40 kPa vacuum level and 90 cycles/min pulsation rate. Again, in experiments carried out in a mid-line milking machine, Fernandez et al. [11], found that the use of 44 kPa compared to 42 kPa (both combined with 120 cycles/min pulsation rate and 60% pulsator ratio) offered higher milk flow during milking. Notwithstanding these benefits, the use of high vacuum levels in both studies entailed an increase in the teat thickness variation after milking, with possible consequences for udder health in the long term, especially, as the authors pointed out, in goat herds with two milkings per day. Mid-line milking machines used to be set up with a higher system vacuum level to avoid an excessive vacuum drop during the high flow phase of milk extraction [14]. This can entail an excessive vacuum level in the teat end during low milk flow phase. On the other hand, low-line milking machines have shown more stable vacuum at the teat end [14]. Thus, it can be deduced that the optimal milking parameters for mid-level and low-level pipes milking machines could be different. 

The aim of the following experiments, both in mid-level and low-level milk line milking machines, was to find the best combination of system vacuum (36 or 40 kPa), pulsation rate (90 or 120 pulse/min) and pulsator ratio (50 or 60%) in the machine milking of Murciano-Granadina goats on variables regarding milking fractioning, milking efficiency, mammary gland health status and animal welfare.

## 2. Materials and Methods

### 2.1. Facilities and Animal Handling

The experiments described in this article were developed at the Experimental and Teaching Farm of the Escuela Politécnica Superior de Orihuela (EPSO) of the Universidad Miguel Hernández de Elche (UMH). The Institutional Animal Care and Use Committee (IACUC) of UMH approved the referenced Animal Use (UMH.DTA.JDS.001.09). Murciano-Granadina goats were kept in a free-stall barn with cereal straw litter and access to outdoor sunny patios. After parturition, goat kids were fed with artificial milk and goats were milked once a day, as usual in the southeast region of Spain. Adult goats were fed according to their productive status with alfalfa hay and cereal mixture. 

Milking facilities were installed in a parlor with two platforms. A low-line milking machine was set up in one of the platforms (1 × 12 × 12) and a mid- line milking machine (120 cm upper milking platform) was equipped to use two platforms (2 × 12 × 12). Both line heights are the most frequently installed in new parlors. These milking machines were similarly equipped with TopFlow clusters (Gea Farm Technologies, Bönen, Germany) and electronic pulsators (StimoPuls Apex M, GeaFarm Technologies, Bönen, Germany). Automatic vacuum shut off system (Lactoflow, GeaFarm Technologies, Bönen, Germany) was set for a minimum milking time of 50 s in the low-line milking machine and 60 s in the mid-line milking machine and to interrupt the vacuum at the 150 g/min of milk flow threshold, with a delay time of 10 s at the end of the milking.

Milking routine during the experiments consisted of cluster attachment, machine milking and cluster detachment by automatic vacuum shut off system. Additionally, during the milking efficiency samplings, extraction of hand stripping milk, injection of 4 IU of oxytocin (Dalmatocina^®^, Fatro Ibérica, Barcelona, Spain), and extraction of residual milk by hand were performed. Finally, both on normal days and on sampling days, teat disinfection with a chlorine solution was carried out.

### 2.2. Experimental Design

Two experiments of one-month duration were performed in Latin square design, one of them in the mid-level milking machine and one in the low-line milking machine. In each experiment, 2 vacuum levels (36 and 40 kPa), two pulsation rates (90 and 120 cycles/min) and two pulsator ratios (50 and 60%) were used. Thus, for each pipeline height there were eight possible treatments (vacuum level-pulsation rate-pulsator ratio):

- 36 kPa—90 cycles/min—50%;
- 36 kPa—120 cycles/min—50%;
- 36 kPa—90 cycles/min—60%;
- 36 kPa—120 cycles/min—60%;
- 40 kPa—90 cycles/min—50%;
- 40 kPa—120 cycles/min—50%;
- 40 kPa—90 cycles/min—60%
- and 40 kPa—120 cycles/min—60%.

From a group of 150 Murciano-Granadina goats, 88 were selected. The selected animals were free of intramammary infection, had machine milk yield higher than 1 kg, milking duration lower than 6 min (see Section 2.3.1), and 90 ± 10 days in milk. The goats were distributed into eight similar groups in 8 different pens (same number of treatments) according to their parturition, machine milk yield and milking duration.

Each of the eight groups of animals was milked according to one of the above described treatments for one week. Monday and Tuesday were used to accustom the animals to the new treatment. On Wednesday, milking efficiency variables were recorded and milk cortisol samples taken. On Thursday, teat end scans were carried out. On Friday, samples of the mammary gland sanitary status variables were taken. On Saturday and Sunday, animals were milked according to their treatment. Once all the controls had been carried out, animals were switched to another treatment to continue the experiment.

### 2.3. Variables Analyzed

#### 2.3.1. Milking Efficiency

Lactocorder^®^ devices (Lactocorder, Balgach, Switzerland) were installed in the long milk tube between the main pipe and the automatic vacuum shut off valves to record machine milk yield (MM, kg) and average (kg/min) and maximum (kg/min) milk flows during the main milking phase. Milking duration (MD, min) was registered using a digital chronometer (HS–70W, Casio^®^, Tokyo, Japan) and was the time elapsed between cluster attachment and the automatic vacuum shut off. Hand stripping milk (HSM, g) and residual milk (RM, g) were recorded with a digital device to ±1 g precision (Zanussi^®^, Alcobendas, Spain).

#### 2.3.2. Mammary Gland Health Status

Bacteriological cultures were performed during the pre-experimental period to select the 88 goats enrolled in the experiment. Somatic cell counts were carried out both during the pre-experimental period and each week during the experimental periods (one per treatment). The procedure during the experiments included a 5 mL sampling of each mammary gland for somatic cell counts that was performed manually prior to machine milking. Later, forestripping and disinfection with 70% ethanol of each teat was performed. Then, another 5 mL of each mammary gland were taken for the bacteriological cultures. Somatic cell count samples were analyzed at the interprofessional dairy laboratory (LICOVAL, Valencia, Spain) using a Fossomatic 5000 device (Foss, Hillerød, Denmark). Bacteriological cultures were performed by seeding 20 μL of milk onto blood agar plates (5% sheep blood, Biomerieux, Lyon, France). The plates were incubated aerobically at 37 °C and examined at 24, 48, and 72 h. It was considered that glands in which milk had more than 5 colonies in the bacteriological cultures or more than 1,500,000 somatic cells/mL, suffered intramammary infection and these goats were not included in the experiment. Somatic cell count values of each gland during the experimental period were transformed into their logarithms in base 10 (log_10_RCS). 

#### 2.3.3. Milk Cortisol and Free Fatty Acids

Lactocorder devices were programmed during the milking efficiency tests of each goat to take representative samples of 50 mL during the individual milking. The samples were kept at 4 °C for less than 4 h. Later, 10 mL aliquots of milk samples were centrifuged (2000G) for 20 min at 4 °C to remove the fat from the top, and, later, an additional 10 μL of azidiol were added to the samples to conserve them for subsequent analysis. These skimmed milk samples were analyzed at the Endocrinology Laboratory of the Veterinary Faculty of the Universidad Complutense (Madrid, Spain). Cortisol was extracted from the skimmed milk samples according to the procedures of Hagen et al. [15]. Then, an enzyme immunoassay to determine cortisol in cattle and validated for goats [16,17] was used for the analysis of cortisol in skimmed milk samples. The quantification of free fatty acids in milk was carried out according to the reference method IDF Standard 50B/1985 published in the Bulletin of the international dairy federation (FIL-IDF, 1991 [18]). The results were expressed in g of oleic acid per 100 g of fat (FFA value (meq/liter) × 28.2/FAT content (g/L).

#### 2.3.4. Teat End Status

Teat end status was evaluated using an ultrasonography device (Agroscan AL, ECM, Noveko International Inc., Angoulême, France) on both teats of the goats before and after the milking, following the procedures of Díaz et al. [18]. The ultrasound scans were analyzed using ECOPEZÓN software (Universidad Miguel Hernández de Elche, Elche, Spain) to obtain the values of the following variables as previously reported by Díaz et al. [19]: increase in teat wall thickness (ITWT%), increase in teat wall area (ITWA, %) and increase in teat end wall area (ITEWA, %). 

#### 2.3.5. Vacuum Level Variables

Vacuum levels and pulsation tests were performed with a Pulsotest Comfort (GeaFarm Technologies, Bönen, Germany) device (response rate of 1500 kPa/s when directly connected to the pulsator [20] and set at a sampling rate frequency of 166.7 Hz) and a NOP needle (Medition Precision SL, Zaragoza, Spain) connected to the short milk tube. Measuring started 20 s after cluster attachment and lasted for 60 s of the main milking phase. Variables recorded were maximum vacuum level during the main milking phase (kPa), minimum vacuum level during the main milking phase (kPa) and average vacuum level during the main milking phase (kPa). Vacuum drop during the main milking phase (kPa) was calculated as the difference between maximum and minimum vacuum level during the main milking phase.

#### 2.3.6. Statistical Analysis

SCC (somatic cell count) values were transformed into their log_10_ (log_10_SCC) to normalize the distribution [21]. 

The data from both experiments were introduced and analyzed on statistical software SAS, 9.2., 2012 (SAS Institute Inc., Cary, NC, USA). 

For each experiment (low-level pipes milking machine and mid-level pipes milking machine) the relationship of the variables related to milking fractioning (machine milk yield, hand stripping milk and residual milk), milking efficiency (milking duration, maximum and average milk flows), teat end-status (increase in teat wall thickness, increase in teat wall area and increase in teat end wall area), log_10_SCC and milk cortisol level with the treatments was studied (see Section 2.2) using a general mixed model (Proc. Glimmix, SAS 9.2., 2012). Machine milk yield was added as covariable for milking duration analyses. Fixed effects included in the model were system vacuum (36 and 40 kPa), pulsation rate (90 and 120 cycles/min), pulsator ratio (50 and 60%) and sampling day (1 to 8). The animal was considered the random term for every variable studied, and in order to consider the repeated measurements over time for the same goat, a “compound symmetry” covariance structure was also included in the previous model.

Additionally, the effect of the treatment (combination of system vacuum, pulsation rate and pulsator ratio) on the vacuum level variables (average vacuum level and vacuum drop) was independently analyzed for low-level and mid-level pipes milking machines, using a general linear mixed model (Proc. GLM, SAS, 9.2., 2012).

## 3. Results

### 3.1. Experiments in Mid-Level Milkline

Results showed that the use of 40 kPa tended to offer better milking fractioning (relation between machine milk yield, hand stripping milk and residual milk) results than 36 kPa. The treatments with 40 kPa provided higher values of MM and lower values of HSM. When using 120 cycles/min pulsation rate combined with 50% pulsator ratio and 90 cycles/min pulsation rate combined with 60% pulsator ratio, the elevation of the system vacuum entailed significantly higher values of MM (up to 0.23 kg). Moreover, all the treatments that include 40 kPa offered significantly lower values of HSM than the corresponding treatments with 36 kPa, with maximum differences of 92 g between 40 kPa-90 cycles/min-60% and 36 kPa-90 cycles/min-60%. A similar effect of the elevation of system vacuum was observed in RM, with significant differences when 90 cycles/min-50%, 25 g, and 90 cycles/min-60%, 22 g, were employed. Regarding milking efficiency, for every combination of pulsation rate and pulsator ratio the elevation of vacuum offered significantly higher values of maximum and average milk flows. Moreover, for every combination of pulsation rate and vacuum system, the elevation of pulsator ratio also enhanced vaules of maximum and average milk flows. No effects were observed in any variable when pulsation rate was 90 cycles/min or 120 cycles/min (Table 1).

Regarding variables related to teat end status, the use of 40 kPa showed higher values of ITWT for every combination of pulsation rate and ratio. This fact also occurred in nearly every combination of pulsator ratio and pulsation rate (except 120 cycles/min and 50%) in the ITWA variable. Moreover, when system vacuum was 40 kPa, raising the pulsation rate from 50% to 60% also enhanced the values of ITWA. On the other hand, the highest ITEWA values were found when 40 kPa and 60% pulsator ratio were combined (Table 1).

No effects of the different treatments were observed in the sanitary status of the mammary gland. Regarding concentration of cortisol in the harvested milk, there were differences between treatments due to variations in pulsation rate or ratio, but hardly explained, as in some cases an elevation or these parameters entailed an increase in cortisol concentration and in other cases entailed a reduction. However, the most relevant result was that for every combination of pulsation rate and pulsator ratio, the use of 40 kPa instead 36 kPa led to a significant elevation of the concentration of the milk cortisol values (Table 1).

Regarding values of the vacuum level variables, the most relevant result was that the use of combinations of 40 kPa system vacuum, 120 cycles/min pulsation rate and 60% pulsator ratio entailed deeper vacuum drops than the combinations of 36 kPa system vacuum and 50% pulsator ratio with 90 or 120 cycles/min (Table 1).

### 3.2. Experiments in Low-Level Milkline

Results of this experiment revealed that milking fractioning was not affected by the treatments employed. Thus, no differences were found between combinations of system vacuum, pulsation rate and ratio, either in the MM variable or in HSM. On the other hand, regarding RM, differences between two specific treatments (36 kPa-90 cycles/min-50% and 40 kPa-120 cycles/min-60%) were found, but these differences were scarce (21 g) and hardly explained by any of the parameters employed. Regarding milking efficiency, the use of a system vacuum of 40 kPa or a pulsator ratio of 60% tended to reduce milking duration. Nevertheless, differences were found only when both mentioned conditions were combined (40 kPa-90 cycles/min-60% offered a reduction on milking duration of 0.59 min compared to 36 kPa-90 cycles/min-50%). A similar situation occurred with maximum milk flow (40 kPa-90 cycles/min-60% offered an enhancement of 0.3 kg/min compared to 36 kPa-90 cycles/min-50%) and average milk flow (40 kPa-120 cycles/min-60% showed a difference of 0.23 kg/min compared to 36 kPa-90 cycles/min-50%). No effects were observed in any variable when pulsation rate was switched from 90 cycles/min to 120 cycles/min.

Regarding teat end status, no differences between treatments were found in the ITWA and ITEWA variables. Conversely, when 120 cycles/min pulsation rate was combined with 50% or 60%, the elevation of system vacuum from 36 to 40 kPa showed higher values of ITEWA (Table 2). 

As observed in the experiments performed in mid-level milk line, no effect of the different treatments was observed in the somatic cell count. It was also observed that every treatment employing 40 kPa offered higher values of milk cortisol than those employing 36 kPa. No differences between treatments were found in the vacuum level variables.

## 4. Discussion

Results found in the variables related to milking fractioning, milking efficiency, vacuum level at the teat end during milking, teat end status, sanitary status of the mammary gland and milk cortisol are similar to those of other experiments carried out in this goat breed [22,23]. 

According to the results found in these experiments, in mid-line level milking machines, the use of 40 kPa system vacuum and 60% pulsator ratio, indifferently combined with 90 or 120 cycles/min/min pulsation rate offered the best performance in terms of milking fractioning and milking efficiency. On the other hand, in low-level milking machines, despite the benefits found with the same combination of milking parameters in milking efficiency (milk flows and milking duration), milking fractioning was not affected. At both height levels it can be concluded that the use of 40 kPa improved milking efficiency. Nevertheless, the question remains of whether it is worth the use of high vacuum level (40 kPa), as in the two experiments carried out, both teat end status variables and milk cortisol proved, higher discomfort for the animals. This discomfort was locally found in the teat end and at a systemic or physiological level. Therefore, decisions when setting a milking machine must be taken carefully depending on the height of the milk line pipes and even on the objectives of each farm, prioritizing milking efficiency or animal health and welfare. This situation is especially relevant when milking in low-line level milking machines. At this height level, the use of 36 kPa system vacuum combined with 60% pulsator ratio achieved similar results for milking fractioning to any combination with 40 kPa, decreasing milk cortisol levels and effects on teat end status. In the literature review there are no similar studies in this goat breed using low-line level milking machines and comparing different system vacuum levels. Results of previous trials [11,12] that recommend using 40 kPa or higher vacuum system level may have led to farmers and milking machine manufacturers to milk with this system vacuum level. The results of the present paper justify that, when using a low-level line milking machine, milking with a system vacuum level higher than 36 kPa would be justified when the aim is milking efficiency. If milking duration is not a limitation (milking duration difference between 36 kPa and 40 kPa was 29 s) the use of 36 kPa could be justified to avoid unnecessary alterations of the teat end status and elevations of the milk cortisol. It can be hypothesized that, when milking in a low-level machine, the use of intermediate values, such as 38 kPa, would achieve a balance between milking efficiency and animal health and welfare. 

In the two experiments performed, the use of 50% pulsation rate did not involve any advantages but a reduction of the milking efficiency. Similar to other studies [13], the 120 cycles/min pulsation rate did not improve milking efficiency when using 40 kPa. This fact is consistent with the similar values of average vacuum level shown in every possible combination with system vacuum or pulsator ratio. In the experiments carried out by Fernandez et al. [13], the use of 120 cycles/min was only advantageous when using 42 kPa system vacuum. However, as these authors pointed out and showed in the present experiments, this elevation of the system vacuum increased the teat oedema, potentially entailing risks for the mammary gland health status. As the cited authors remarked, the udder morphology, specifically the sphincter strength, is one of the limitations of milking efficiency, as it is inversely related to the values of milk flows. In this sense, these elevations of the system vacuum level should not be the solution to the milking slowness found in this breed. This is particularly important at the end of the milking, as the less milk there is in the milk tubes, the higher the vacuum at the teat end, with the consequences for udder health mentioned above. In fact, recent studies in dairy cows [24] have tested a prototype to decrease the system vacuum during low milk flow phases due to its advantages. In this sense, in goats, the possible benefits in terms of animal health and welfare of the use of low-level line milking machines set up with lower levels of system vacuum if milking fractioning or milking efficiency are not affected must not be ignored. 

In the two experiments carried out, worse values of milk cortisol and teat end status were not related to higher somatic cell count values. This result must not be interpreted as a general conclusion for all Murciano-Granadina goats farms. This work was performed in a once-a-day milking frequency, milking was carried out by experienced workers and with the use of an automatic vacuum shut off system. Conversely, as milking conditions as previously described are different (two milkings per day, rough machine stripping or detaching of the teat cups without previous vacuum shutting off), the use of high system vacuum levels could increase the effects on the teat end status more than in the present experiments, even not reaching the minimum time for teat end recuperation [25], entailing a worsening of the values of variables related to mammary gland health status, somatic cell count or even incidence of mastitis. 

The milk cortisol values (from 0.39 to 1.28 ng/mL) are similar to those found in previous experiments carried out by this research group in similar conditions [23,26]. Milk cortisol has been described as a suitable tool to determine if farming conditions (feeding, milking, housing) cause stress to goats [26]. In the present experiments, a correlation between the effect of machine milking (vacuum) on teat end status and cortisol has been proven. It has been previously described that excessive system vacuum can alter teat end status [3]. In the present experiments, this effect was observed more in mid-level machine experiment than in the low-level machine. In these experiments, this situation coincides with values of milking fractioning, establishing an inverse relation between milking efficiency on the one hand and teat status and cortisol on the other. As milking parameters are programmed to improve milking efficiency, when using a higher system vacuum (40 kPa) increased milk cortisol and teat end changes after milking are found. So, although milking duration increases, if overmilking is not practiced as in the experiments performed by Peris et al. [27] and Alejandro et al. [28], a longer milking duration, as observed in the treatments with 36 kPa, is not associated with greater changes in teat end status.

Despite the cited milk cortisol values, there was no inhibitory effect of the oxytocin release during milking, as variations in residual milk values were not related to changes in the milking parameters. As previously reported in different studies and unlike in cows, milking of goats can begin without previous stimulation [29]. Thus, in goats, milk flows from the very beginning of the milking after the cluster attachment and during the main milking phase [30]. Presence of milk in the tubes lowers vacuum at the surface of the teat end, especially in mid-line machines [22,31]. It is possible that goats only feel high system vacuum as a stressful stimulus at the end of milking when oxytocin response has been developed and residual milk has already been released from alveoli to the cisternal compartment in the mammary gland. This system vacuum when milk flow has decreased creates discomfort at local level, increasing the teat end oedema, and at systemic level, resulting in higher milk cortisol levels. 

Although the trials carried out in mid-line and low-line were performed as different experiments, vacuum level values measured at the teat end confirmed the results of Manzur et al. [14], who observed that the use of mid-level line milking machines is related to an increase in the vacuum drop during milking.

## 5. Conclusions

Depending on milk line height, milking parameters affected milking efficiency, milking fractioning, teat end-status and milk cortisol of Murciano-Granadina goats differently. In milking machines equipped with mid-level pipes, the use of 40 kPa system vacuum, 60% pulsator ratio and 90 or 120 cycles/min pulsation rate reached optimum milking fractioning and efficiency without affecting in short term the health status of the animals. In milking machines fitted with low-level pipes, the use of 40 kPa system vacuum, 60% pulsator ratio and 90 or 120 cycles/min pulsation rate offered an advantage in terms of milking efficiency. Nevertheless, a similar combination with 36 kPa did not entail worse milking fractioning values and provided better values of teat end status and cortisol level. Although future experiments are needed to confirm this hypothesis, when milking in a low-level machine, the use of intermediate values, such as 38 kPa, could achieve a balance between milking efficiency and animal health and welfare. These milking machine settings must be tested in a long-term experiment to determine their effects on milk yield throughout lactation, mammary gland health status and milk composition.

## Figures and Tables

**Table 1:** Effect of the treatment (combinations of system vacuum, pulsation rate and pulsator ratio) on variables related to milking fractioning, milking efficiency, teat end status, milk composition, animal welfare and vacuum measured in the short milk tube in Murciano-Granadina goats milked in mid-line pipes (least square means ± standard error of the mean).

Variable	36 kPa 120 p/m 50%	36 kPa 120 p/m 60%	36 kPa 90 p/m 50%	36 kPa 90 p/m 60%	40 kPa 120 p/m 50%	40 kPa 120 p/m 60%	40 kPa 90 p/m 50%	40 kPa 90 p/m 60%	SEM	SL
MM (kg)	1.33 ^bc^	1.37 ^abc^	1.34 ^abc^	1.25 ^c^	1.50 ^a^	1.44 ^ab^	1.44 ^ab^	1.48 ^a^	0.09	<0.05
HSM (g)	230 ^a^	223 ^a^	253 ^a^	256 ^a^	159 ^b^	175 ^b^	168 ^b^	164 ^b^	23	<0.05
RM (g)	85 ^ab^	80 ^abc^	97 ^a^	94 ^a^	74 ^bc^	64 ^c^	72 ^bc^	72 ^bc^	9	<0.05
MD (g)	3.13 ^ab^	2.78 ^bc^	3.39 ^a^	2.97 ^abc^	3.08 ^ab^	2.59 ^bc^	3.01 ^abc^	2.52 ^c^	0.25	<0.05
MaxFlow (kg/min)	0.96 ^cd^	1.17 ^ab^	0.90 ^d^	1.09 ^bc^	1.11 ^b^	1.28 ^a^	1.11 ^b^	1.30 ^a^	0.07	<0.05
AvgFlow (kg/min)	0.58 ^e^	0.66 ^cd^	0.58 ^e^	0.63 ^de^	0.71 ^bc^	0.78 ^ab^	0.69 ^cd^	0.80 ^a^	0.04	<0.05
ITWT (%)	34.6 ^d^	31.2 ^d^	44.0 ^c^	44.9 ^c^	43.0 ^c^	53.7 ^b^	46.5 ^c^	66.4 ^a^	4.0	<0.05
ITWA (%)	57.3 ^bcd^	45.4 ^d^	40.5 ^d^	51.2 ^cd^	68.3 ^abc^	85.9 ^a^	69.4 ^abc^	71.2 ^ab^	9.5	<0.05
ITEWA (%)	21.6 ^b^	13.1 ^c^	16.2 ^bc^	15.4 ^c^	20.7 ^b^	31.2 ^a^	21.2 ^b^	33.4 ^a^	2.6	<0.05
Log_10_SCC	2.57	2.55	2.51	2.57	2.47	2.54	2.50	2.54	0.09	ns
Cortisol (ng/mL)	0.83 ^d^	0.89 ^cd^	0.81 ^d^	0.78 ^d^	0.97 ^bc^	1.21 ^a^	1.28 ^a^	0.97 ^bc^	0.11	<0.05
FFA (meq/mL)	0.61	0.58	0.54	0.57	0.55	0.53	0.61	0.62	0.04	ns
MaxVacLevel (kPa)	33.9 ^a^	34.0 ^a^	34.6 ^a^	34.6 ^a^	37.7 ^b^	38.2 ^b^	38.2 ^b^	38.5 ^b^	0.5	<0.05
AvgVacLevel (kPa)	31.0 ^a^	30.9 ^a^	31.5 ^a^	31.5 ^a^	34.7 ^b^	34.4 ^b^	34.8 ^b^	35.1 ^b^	0.5	<0.05
Vacuum Drop (kPa)	6.7 ^b^	7.8 ^ab^	7.6 ^b^	7.8 ^ab^	6.4 ^b^	9.0 ^a^	7.3 ^b^	8.8 ^ab^	1.1	ns

MM: machine milk yield; HSM: hand stripping milk; RM: residual milk; MD: milking duration; MaxFlow: maximum milk flow during the main milking phase; AvgFlow: average milk flow during the main milking phase; ITWT: increase in teat wall thickness; ITWA: increase in teat wall area; ITEWA: increase in teat end wall area; Log_10_ of the somatic cell count values; Cortisol: milk cortisol; FFA: free fatty acids in milk. MaxVacLevel: maximum vacuum level at the short milk tubes during the main milking phase; AvgVacLevel: average vacuum level at the short milk tubes during the main milking phase. Values in the same line with different letters (^a^^–^^e^) differ at *p* < 0.05; ns: not significant, *p* > 0.05. SEM: standard error of the mean. SL: significance level.

**Table 2:** Effect of the treatment (combinations of system vacuum, pulsation rate and pulsator ratio) on variables related to milking fractioning, milking efficiency, teat end status, milk composition, animal welfare and vacuum measured in the short milk tube in Murciano-Granadina goats milked in low-line pipes (least square means ± standard error of the mean).

Variable	36 kPa 120 p/m 50%	36 kPa 120 p/m 60%	36 kPa 90 p/m 50%	36 kPa 90 p/m 60%	40 kPa 120 p/m 50%	40 kPa 120 p/m 60%	40 kPa 90 p/m 50%	40 kPa 90 p/m 60%	SEM	SL
MM (kg)	1.57	1.57	1.49	1.49	1.62	1.59	1.61	1.55	0.11	ns
HSM (g)	191	184	197	223	205	206	184	207	22	ns
RM (g)	94 ^ab^	95 ^ab^	108 ^a^	100 ^ab^	86 ^ab^	79 ^b^	83 ^ab^	82 ^ab^	14	<0.05
MD (g)	3.01 ^ab^	2.92 ^ab^	3.02 ^a^	2.91 ^ab^	2.81 ^abc^	2.57 ^bc^	2.81 ^abc^	2.43 ^c^	0.21	<0.05
MaxFlow (kg/min)	0.98 ^cd^	1.04 ^bc^	0.87 ^d^	1.03 ^bc^	1.07 ^abc^	1.20 ^a^	1.00 ^cd^	1.17 ^ab^	0.08	<0.05
AvgFlow (kg/min)	0.61 ^bc^	0.69 ^b^	0.57 ^c^	0.67 ^bc^	0.71 ^ab^	0.8 ^a^	0.69 ^b^	0.76 ^ab^	0.05	<0.05
ITWT (%)	40.7 ^b^	31.1 ^c^	43.4 ^ab^	43.3 ^b^	52.0 ^a^	48.9 ^a^	40.4 ^b^	43.1 ^b^	4.3	<0.05
ITWA (%)	53.6	50.2	49.6	53.1	49.8	60.7	60.5	57.2	9.5	ns
ITEWA (%)	20.2	21.9	19.4	21.5	20.9	21.3	20.3	18.3	2.5	ns
Log_10_SCC	2.52	2.46	2.50	2.49	2.33	2.34	2.37	2.38	0.11	ns
Cortisol (ng/mL)	0.50 ^b^	0.39 ^b^	0.51 ^b^	0.51 ^b^	0.82 ^a^	0.81 ^a^	0.80 ^a^	0.72 ^a^	0.08	<0.05
FFA (meq/mL)	0.37	0.44	0.39	0.53	0.49	0.46	0.51	0.42	0.08	ns
MaxVacLevel (kPa)	36.1 ^a^	35.9 ^a^	35.9 ^a^	36.0 ^a^	39.6 ^b^	39.6 ^b^	39.5 ^b^	39.6 ^b^	0.2	<0.05
AvgVacLevel (kPa)	35.3 ^a^	35.3 ^a^	35.2 ^a^	35.3 ^a^	38.9 ^b^	38.8 ^b^	38.8 ^b^	38.8 ^b^	0.2	<0.05
Vacuum Drop (kPa)	2.2	1.9	2.3	2.0	1.8	2.4	2.4	2.4	0.2	ns

MM: machine milk yield; HSM: hand stripping milk; RM: residual milk; MD: milking duration; MaxFlow: maximum milk flow during the main milking phase; AvgFlow: average milk flow during the main milking phase; ITWT: increase in teat wall thickness; ITWA: increase in teat wall area; ITEWA: increase in teat end wall area; Log_10_ of the somatic cell count values; Cortisol: milk cortisol; FFA: free fatty acids in milk. MaxVacLevel: maximum vacuum level at the short milk tubes during the main milking phase; AvgVacLevel: average vacuum level at the short milk tubes during the main milking phase. Values in the same line with different letters (^a^^–^^c^) differ at *p* < 0.05; ns: not significant, *p* > 0.05. SEM: standard error of the mean. SL: significance level.

## Data Availability

The data presented in this study are available on request from the first author.
